# Unique microRNA expression profiles in plasmic exosomes from intrahepatic cholestasis of pregnancy

**DOI:** 10.1186/s12884-023-05456-1

**Published:** 2023-03-07

**Authors:** Yao Kong, Yongchi Zhan, Daijuan Chen, Xixi Deng, Xinghui Liu, Tingting Xu, Xiaodong Wang

**Affiliations:** 1 Department of Obstetrics and Gynecology, West China Second University Hospital, Sichuan University, Chengdu, Sichuan province, China; 2 Key Laboratory of Birth Defects and Related Diseases of Women and Children (Sichuan University), Ministry of Education, Sichuan, 610041 China

**Keywords:** Exosomes, microRNA, Hsa-miR-940, Hsa-miR-636, Hsa-miR-767-3p, Biomarker, Intrahepatic cholestasis of pregnancy (ICP)

## Abstract

**Background:**

Intrahepatic cholestasis of pregnancy (ICP) is strongly associated with an increased risk of adverse perinatal outcomes. Total bile acid (TBA) levels in the late second or third trimester are a major factor in the diagnosis. Here, we sought to establish the miRNA expression profile of plasm exosomes of ICP and identify possible biomarkers for the diagnosis of ICP.

**Methods:**

This case–control study involved 14 ICP patients as the experimental group and 14 healthy pregnant women as the control group. Electron microscopy was used to observe the presence of exosomes in plasma. Nanosight and Western blotting of CD63 was used to assess exosome quality. Among them, three ICP patients and three controls were used for isolation plasmic exosome and preliminary miRNA array analysis. The Agilent miRNA array was utilized to dynamically monitor the miRNA expression in plasmic exosomes of included patients in the first trimester(T1), second trimester (T2), third trimester (T3), and delivery (T4). Then, Quantitative real-time Polymerase chain reaction was used to identify and validate differentially expressed miRNAs in plasma-derived exosomes.

**Results:**

The expression levels of hsa-miR-940, hsa-miR-636, and hsa-miR-767-3p in plasma-derived exosomes of ICP patients were significantly higher than those of healthy pregnant women. Besides, these three miRNAs were also significantly up-regulated at the plasma, placental, and cellular levels (*P* < 0.05). The diagnostic accuracy of hsa-miR-940, hsa-miR-636, and hsa-miR-767-3p was further evaluated by the ROC curve, the area under the curve (AUC) values for each were 0.7591, 0.7727, and 0.8955, respectively.

**Conclusions:**

We identified three differentially expressed miRNAs in the plasma exosomes of ICP patients. Hence, hsa-miR-940, hsa-miR-636, and hsa-miR-767-3p may be potential biomarkers for enhancing the diagnosis and prognosis of ICP.

**Supplementary Information:**

The online version contains supplementary material available at 10.1186/s12884-023-05456-1.

## Introduction

Intrahepatic cholestasis of pregnancy (ICP) is the most common pregnancy-specific liver disease, which usually manifests in the middle and late trimesters of pregnancy [1]. Clinical signs include skin pruritus and jaundice, while biochemical manifestations include increased total bile acids (TBA) levels and impaired liver function. The condition progresses such that the aforementioned symptoms and biochemical abnormalities are relieved rapidly after delivery [2]. The etiology and pathogenesis of ICP are relatively complex and have not been fully elucidated at present. Several studies have suggested that it may be related to factors such as inflammation, apoptosis, oxidative stress, lipid metabolism, cell growth, and immune response [3, 4]. Although ICP is reversible in pregnant women, it is associated with an increased risk of perinatal complications including spontaneous preterm birth, meconium-staining amniotic fluid, fetal distress, and sudden intrauterine death [5]. ICP is an exclusionary diagnostic. Currently, the diagnosis of it mainly depends on clinical symptoms and TBA concentration in the second trimester or third trimester. Finding new diagnostic and prognostic ICP biomarkers is crucial since ICP diagnosis has certain limits in clinical work.

MicroRNAs (miRNAs) are non-coding single-stranded RNA molecules encoded by endogenous genes (including animal, plant, some single-celled organisms and viral genomes) with a length of about 22 nucleotides. They do not have an open reading frame (ORF) and do not encode proteins, but can combine with specific complementary sequences in the non-coding region of the 3 '- UTR end of the mRNA of the target gene, mediate the degradation of mRNA or inhibit the translation of protein, and regulate the expression of genes and proteins at the post-transcription level [6, 7]. Due to the wide range of gene regulation capabilities and tissue specificity of miRNAs, researchers assume that they may play important regulatory functions in various systems, tissues, and organs [8]. Studies have found that some pregnancy-related diseases are often accompanied by abnormal expression of miRNAs in placental tissue and even in the peripheral circulation [9–11]. Recent research has shown that the expression of microRNA is closely associated with the occurrence and progression of several pregnancy-related diseases, such as recurrent spontaneous abortion, gestational diabetes mellitus, gestational hypertension, ICP, etc. [12–14]. In addition, with the continuous accuracy of detection technology and the accessibility of serum microRNAs, circulating microRNAs have emerged as a kind of disease marker with diagnostic and prognostic potential [15].

MicroRNAs can also be actively released into the circulatory system via exosomes in addition to passive secretion [16]. Exosomes are a class of small membrane transporters with a diameter of about 40–100 nm, which are actively secreted from cells into the extracellular microenvironment, and almost all types of cells can secrete exosomes [17]. Exosomes carry proteins, miRNAs, lncRNAs, circRNAs, mRNAs, and their degradation fragments involved in intracellular signal transduction, and participate in the important regulation of cell activities by transmitting relevant signal molecules to adjacent or distant cells, such as those involved in gene expression regulation, survival and reproduction, reproduction and development, angiogenesis, wound healing and other processes [18]. Among them, exosomes in the blood circulation can be absorbed by recipient cells and affect the biological functions of recipient cells [19]. In addition, the circulating exosomal RNAs are resistant to biochemical degradation by ribonuclease and RNase A in serum under an in vitro condition, so exosomal RNAs are more stable than cellular RNAs [20]. The above characteristics enable circulating exosomes to provide a stable source of RNAs, which can be used as a basis for disease diagnosis, prognosis, and treatment, and it is expected to become an early diagnostic marker of various diseases.

At present, the related research on ICP and exosomes is mainly concentrated in the urine, while the related research on circulating exosomes has not yet been conducted. Furthermore, we know that ICP, in clinical practice, is usually diagnosed in the second and third trimesters of pregnancy, and early screening and prevention cannot be achieved. In the present study, we pursued circulating exosomes, isolated from ICP and the time-matched control group in four different periods: the first trimester, the second trimester, the third trimester, and delivery, trying to find biomarkers that can diagnose ICP early. And quantitative reverse transcriptase-polymerase chain reaction (RT-qPCR) assay was used to identify the unique microRNA signals in plasma exosomes from ICP patients after the Agilent miRNA array.

## Methods

### Participants

In this study, we recruited pregnant women who had regular prenatal care and delivered at West China Second Hospital of Sichuan University from January 2020 to December 2021. All these included population followed up their prenatal examination process, and collected maternal plasma during the first trimester (T1), second trimester (T2), third trimester (T3), and delivery (T4). According to the rules of the Chinese Medical Association of Obstetrics and Gynecology branch [21], ICP is diagnosed by increased maternal serum TBA ≧ 10 µmol/L with or without pruritus and quick disappearance after delivery, as well as the elimination of other causes of liver malfunction or itching. The control group included healthy pregnant women who matched gestational age of the ICP group. All samples were from women with singleton pregnancies. Inclusion criteria of the control group: healthy pregnant women with similar gestational weeks as those in the ICP group, who conceived naturally and with no other pregnancy complications, no medical-related complications, and no history of abortion; Exclusion criteria: got liver disease, biliary tract disease, or autoimmune disease before pregnancy; or pregnant with complications such as gestational hypertension and gestational diabetes mellitus, or medical-related complications.

According to the inclusion and exclusion criteria, a total of 28 pregnant women were enrolled in this case–control study, with 14 ICP patients and 14 healthy pregnant women serving as the experimental group and the control group, respectively. Among them, three ICP patients and three controls were used for isolation plasmic exosome and preliminary miRNA array analysis. Besides, Nanosight was used for nanoparticle tracking analysis and Western blotting of CD63 to assess exosome quality. The plasma samples in the ICP group and control group were 10 and 11, respectively. The remaining subjects, including the 8 patients screened for differential miRNAs, were used for subsequent validation. Written informed consents were obtained from patients before the procedure and manuscript publication. This study was approved by the ethical committees at the West China Second University Hospital of Sichuan University.

### Sample collection

During prenatal care and follow-up, blood samples were collected from participants in the first trimester (8–11 weeks), second trimester (22–24 weeks) and third trimester (34–36 weeks), and finally both blood samples and placental tissue were collected at delivery. All participants were instructed to sit still for half an hour and then take 3 ml of fasting elbow venous blood in a sitting position with an EDTA anticoagulation tube between 8:00 and 8:30 am, and centrifuged at 3000 r/min for 10 min after resting for 3–4 h at 4 °C. Then, the supernatant plasma was divided into sterilized enzyme-free EP tubes and frozen at -80 °C for future use. In addition, after the placenta was isolated, a tissue of about 1 cm × 1 cm × 1 cm was cut from the maternal surface of the placenta, washed with saline and placed in sterile enzyme-free EP tubes, and immediately put into liquid nitrogen for freezing and storage.

### Isolation and examination of exosomes from plasma

Plasma samples were obtained and processed within 2 h. The processing procedure involved centrifugation at 2,000 g for 30 min at 4 °C, then aspiratingthe supernatant and centrifuge at 12,000 g for 45 min at 4 °C, continue to aspirate the supernatant and centrifuge at 110,000 g for 70 min at 4 °C, discard the supernatant and resuspend in 100 μL of 1 × phosphate buffered saline (PBS) and store at -80 °C until analysis. Then, according to the method shown in Bang C, et al. [22], the morphology and distribution of the extracted exosomes were measured by Transmission Electron Microscopy and Nanosight, and exosome markers were detected by Western Blot.

### The miRNA microarray analysis

After extraction of total RNA from plasma exosomes, NanoDrop ND-2000 (Thermo Scientific) was used to quantify and Agilent Bioanalyzer 2100 (Agilent Technologies) was applied to detect the integrity of RNA. Then, the total RNA was reverse transcribed into double-stranded cDNA, and cRNA labeled with Cyanine-3-CTP (Cy3) was further synthesized. The labeled cRNA was hybridized with the microarray, and the raw image was obtained by scanning with an Agilent Scanner G2505C (Agilent Technologies) after elution (GEO accession number GSE210764).

### RNA isolation and Quantitative real-time Polymerase chain reaction (qRT-PCR)

Total RNA from tissues and cells was extracted with Trizol reagent (Takara, Code No. 9109) according to the kit instructions. The RNA isolated from each sample was then reverse transcribed into cDNA using the PrimeScript™ RT reagent Kit with gDNA Eraser (Takara, Code No. RR047A). The reaction conditions for reverse transcription were as follows: genomic DNA was removed at 42 °C for 2 min, reverse transcription at 37 °C for 15 min, and reverse transcriptase was inactivated at 85 °C for 5 s. Next, Quantitative real-time PCR was performed using the TB Green® Premix Ex Taq™ II Kit (Takara, Code No. RR820A). The amplification conditions were as follows: pre-denaturation at 95 °C for 30 s, PCR reaction for 40 cycles, denaturation at 95 °C for 5 s, and annealing at 60 °C for 30 s. The relative expression levels were calculated using the 2^−ΔΔCT^Cq method.

### Western blot

Exosome samples were lysed in RIPA lysis buffer (ThermoScientific™, Code No. 89901) and centrifuged at 13,000 rpm for 10 min at 4 °C. Protein concentration was determined and then separated with polyacrylamide gel electrophoresis using 10% SDS-PAGE gels. Proteins were transferred to polyvinylidene difluoride membranes, which were then incubated with 5% bovine serum albumin in Tris-buffered saline for 2 h at room temperature to block nonspecific binding. Membranes were then incubated with the primary antibodies overnight at 4 °C. After three washes, the samples were incubated with goat anti-rabbit secondary antibody for 60 min at room temperature. Finally, the specific proteins were detected using enhanced chemiluminescence (ThermoScientific™, Code No. 34580).

### Cell culture and construction of cholestatic cell model

The human chorionic villus trophoblast cell line (HTR8/SVneo cells) was obtained from the American Type Culture Center (ATCC) and identified by STR Authentication. HTR8/Svneo cells were cultured in 90% RPMI 1640 medium, 1.0% dual antibodies (penicillin and streptomycin), and 10% fetal bovine serum in a complete medium. HTR8/Svneo cells were seeded in sterile 6-well plates and cultured until the cell density reached 70%, and then treated with 100 μmol/L sodium taurocholate (TCA) for 24 h to establish a cholestatic cell model [23].

### Statistical analysis

To analyze raw data from Agilent chips, Feature Extraction software (version10.7.1.1, Agilent Technologies) was used to analyze array of images to extract raw data. Then, Genespring software (version 14.8, Agilent Technologies) was applied toanalyze the raw data and identify differentially expressed miRNAs. And the target genes of differentially expressed miRNAs were the predicted intersections with the miRDB and miRWalk databases. Finally, predictive analysis of the miRDB database was applied to predict the roles of these target genes. Hierarchical Clustering was performed to reveal the distinguishable miRNAs expression pattern among samples. Furthermore, statistical analyses were conducted using Microsoft Excel and GraphPad Prism 8 (https://www.graphpad.com/). The results were presented as the mean ± standard deviation (SD). Differences between two groups were compared by the Student’s t test, while differences among multiple groups were compared by one-way or two-way ANOVA analysis of variance. Meanwhile, receiver operating characteristic (ROC) curve analysis was further used to evaluate the diagnostic value of biomarkers for ICP, and the sensitivity and specificity were obtained by referring to the area under the curves (AUCs), where AUC greater than 0.70 was considered as an acceptable level of discrimination.

## Results

### Clinical characteristics of the included population

The baseline characteristics and perinatal outcomes of 14 patients with ICP and 14 healthy expectant mothers are summarized in Table 1. There was no significant difference in maternal age, body mass index (BMI), and newborn weight between the two groups (*P* > 0.05). However, the levels of TBA, alanine aminotransferase (ALT), and aspartate aminotransferase (AST) were significantly higher in ICP patients than in healthy pregnant women. In addition, ICP patients had significantly lower gestational weeks at delivery compared to healthy controls (Table 1).

**Table 1:** Baseline characteristics of ICP patients and healthy pregnant women

Variable	Con (*N* = 14)	ICP(*N* = 14)	*P*-value
Age(years)	37.4 ± 1.6	37.6 ± 2.9	0.72
Body Mass Index(kg/cm2)	25.5 ± 1.8	26.0 ± 1.9	0.58
TBA(μmol/L)	1.6 ± 0.8	36.1 ± 28.3	0.00*
ALT(U/L)	20.5 ± 20.0	146.9 ± 150.8	0.00*
AST(U/L)	20.1 ± 8.7	117.5 ± 140.0	0.00*
Gestational weeks	39.4 ± 0.4	37.9 ± 1.4	0.00*
Newborn weight (g)	3345 ± 387.2	3105 ± 462.5	0.20

Con, healthy pregnant women; *ICP* ICP patients, *TBA* Total bile acids, *ALT* Alanine aminotransferase, *AST* Aspartate aminotransferase; *P < 0.05

### Analysis and identification of plasma-derived exosomes

**Fig. 1:**
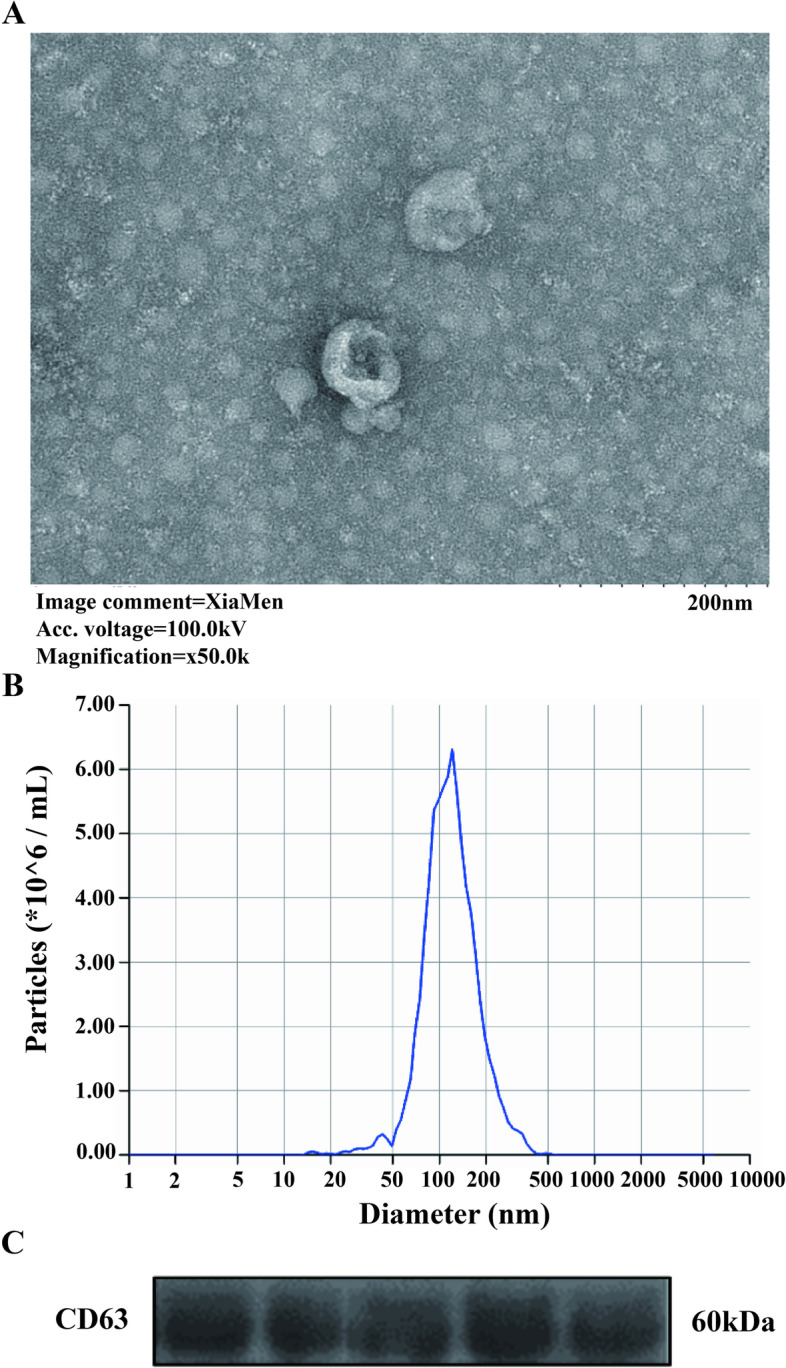
Analysis and identification of plasma-derived exosomes. **A** Electronmicroscopy shows the morphology and particle size distribution of exosomes; **B** Nanosight analysis shows the number of exosomes and the particle size distribution; **C** Western Blot shows the surface marker CD63 expression of exosomes

We isolated plasma exosomes using ultracentrifugation, which is the current gold standard method for exosome extraction. Electron microscopy was used to examine the morphology of plasma exosomes, as seen in Fig. 1A. It was found that the exosomes in the plasma of ICP patients were intact, spherical, and homogeneous in size. Plasma-derived exosomes all expressed the characteristic tetraglycan protein CD63, as well as the expected Nanosight profile (Fig. 1B, C), indicating that plasma-derived exosomes are a suitable sample to observe the differentiated expression of miRNAs in included patients.

### Screening and target gene prediction of differential miRNAs from plasma-derived exosomes

**Fig. 2:**
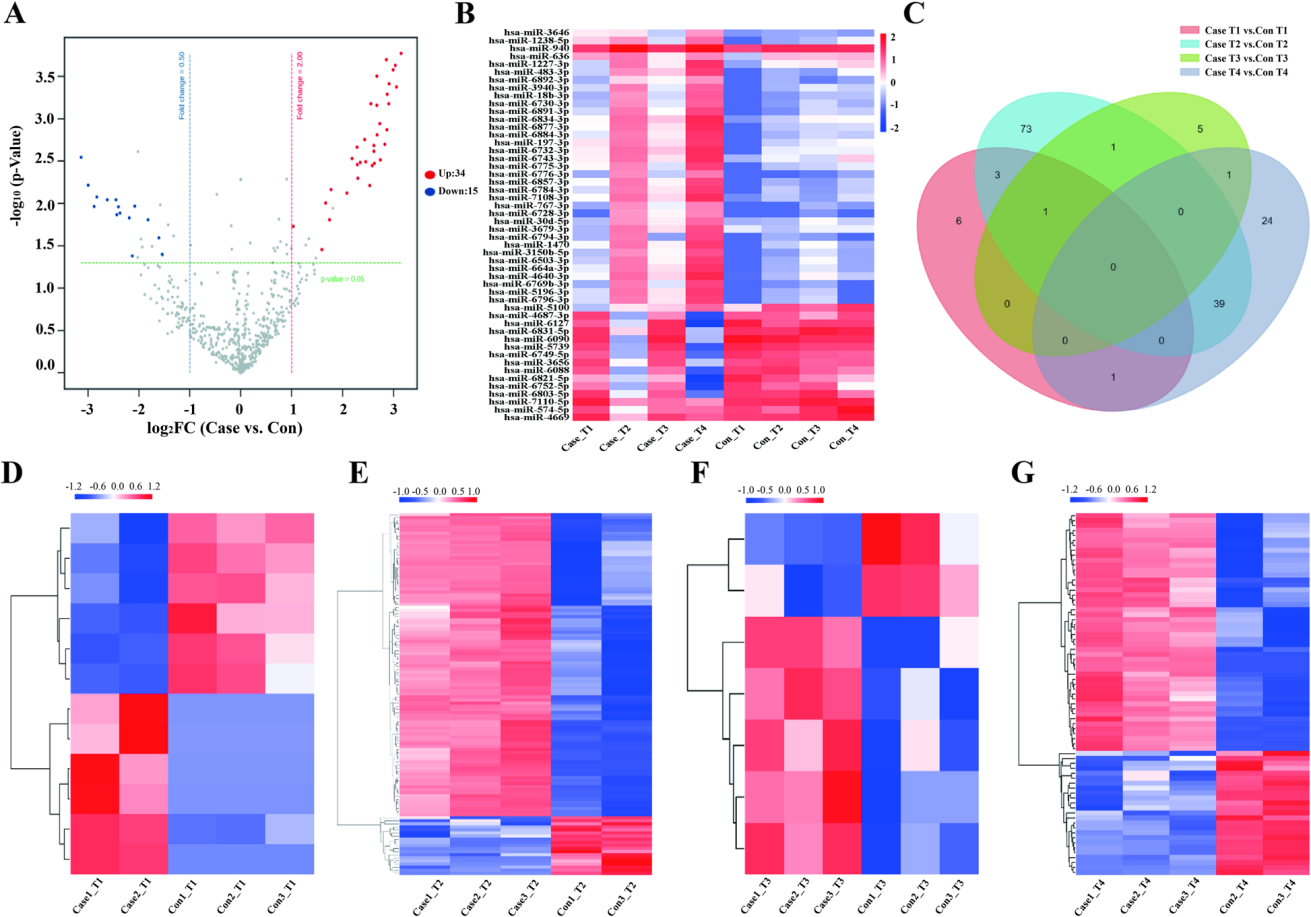
Bioinformatics analysis of differentially expressed plasma-derived exosomal miRNAs. **A** Volcano plot of differentially expressed plasma-derived exosomal miRNAs of ICP patients and healthy pregnant women. The scattered dots in the figure represent individual miRNAs. Gray dots indicate non-significantly differentially expressed miRNAs, red dots indicate significantly up-regulated differential miRNAs, and blue dots indicate significantly down-regulated differential miRNAs; **B** Cluster analysis of differentially expressed plasma-derived exosomal miRNAs of ICP patients and healthy pregnant women. Clustering was performed with log10 (TPM + 1) values, with red color indicating highly expressed miRNAs and blue color indicating lowly expressed miRNAs; **C** Venn diagram of plasma-derived exosomal miRNAs that are differently expressed in ICP patients and healthy pregnant women. Red, blue, green, and purple indicate differentially expressed plasma-derived exosomal miRNAs specific to T1, T2, T3, and T4, respectively; Cluster analysis of differentially expressed plasma-derived exosomal miRNAs in ICP patients and healthy pregnant women during T1 (**D**), T2 (**E**), T3 (**F**) and T4 (**G**). Con, healthy pregnant women; Case, ICP patients; T1, first trimester; T2, second trimester; T3, third trimester; T4, delivery

We elected to use Agilent miRNA microarray analysis technology to detect miRNA levels in exosomes after evaluating the sensitivity of next-generation miRNA sequencing technology applied to plasma-derived exosomal miRNAs. First, we inferred the overall distribution of differential miRNAs in plasma-derived exosomes using volcano plots, and screened for differential miRNAs by assessing both the fold change and corrected *p*-value (qvalue). The default screening condition for differential miRNAs was twofold upregulation or 0.5-fold downregulation, and qvalue < 0.05 when the samples had biological replicates. The results showed that a total of 49 differentially expressed miRNAs were screened, of which 34 miRNAs were up-regulated and 15 miRNAs were down-regulated (Fig. 2A, B). Then, we calculated the number of differential miRNAs obtained by comparing T1, T2, T3, and T4 these four different periods, and made a Venn diagram. The findings revealed thatthe number of miRNAs shared among T1, T2, T3, and T4 was 0, while the numbers of miRNAs unique to T1, T2, T3, and T4 were 6, 73, 5, and 24, respectively (Fig. 2C). Additionally, we further used cluster analysis to compare the number of differential miRNAs in each stage of T1, T2, T3, and T4, respectively, and discovered that there were, respectively, 12, 117, 7, and 73 difference miRNAs at each stage of T1, T2, T3, and T4 (Fig. 2D-F).

**Fig. 3:**
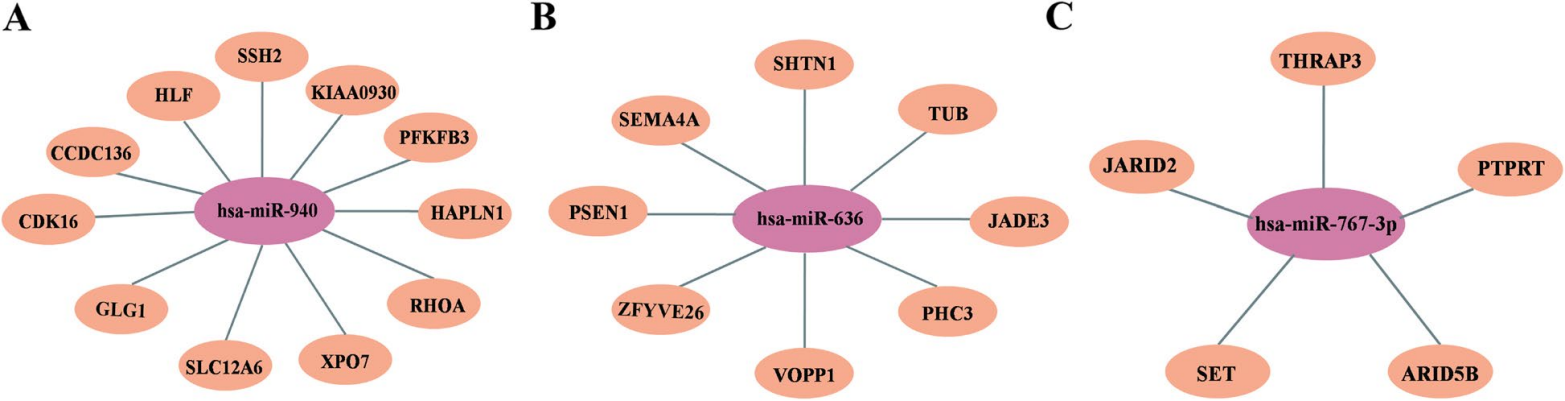
Target genes of differentially expressed plasma-derived exosomal miRNAs. **A** Target genes associated with hsa-miR-940; **B** Target genes associated with hsa-miR-636; **C** Target genes associated with hsa-miR-767-3p

In the meanwhile, we predicted the target genes of exosome-derived differentially expressed hsa-miR-940, hsa-miR-636, and hsa-miR-767-3p. The miRDB database was used to predict all target genes, and Fig. 3 showed the predicted target genes with a miRDB score greater than 90. Target genes associated with hsa-miR-940 included SSH2(92.07), KIAA0930 (94.90), PFKFB3 (94.13), HAPLN1 (92.07), RHOA (90.21), XPO7 (94.48), SLC12A6 (90.80), GLG1 (93.81), CDK16 (90.73), CCDC136 (92.84), and HLF (91.37) (Fig. 3A). Additionally, the target genes for hsa-miR-636 included SHTN1 (94.56), TUB (91.88), JADE3 (91.73), PHC3 (93.14), VOPP1 (90.70), ZFYVE26 (91.05), PSEN1 (91.14), and SEMA4A (94.25) (Fig. 3B). Also, the target genes associated with hsa-miR-767-3p include THRAP3 (91.97), PTPRT (96.82), ARID5B (91.05), SET (91.77) and JARID2 (94.69) (Fig. 3C).

### Validation of differentially expressed plasma-derived exosomal miRNAs at the plasma level

In plasma, miRNAs are not only found in exosomes but can also be present in other vesicles or bound to plasma non-vesicular proteins [24, 25]. To assess whether differential expression of exosomal miRNAs can be detected using whole plasma samples, we compared the expression levels of eight differentially expressed exosomal miRNAs in whole plasma from healthy pregnant women and ICP patients at each stage of T1, T2, T3, and T4. Candidate miRNAs selected for validation included seven differentially expressed miRNAs and one miRNA with no statistically significant differential expression but with a |logFC| of more than twofold (Table 2).

**Table 2:** Differentially expressed serum exosomal miRNAs for validation

Exosome miRNA	Fold change	*P*-value	Change in expression level
hsa-miR-940	2.0412	0.0187	Up
hsa-miR-30d-5p	3.1781	0.0098	Up
hsa-miR-18b-3p	4.8595	0.0022	Up
hsa-miR-767-3p	5.8665	0.0007	Up
hsa-miR-197-3p	6.1149	0.0024	Up
hsa-miR-483-3p	7.2484	0.0002	Up
hsa-miR-664a-3p	7.4840	0.0007	Up
hsa-miR-636	2.2475	0.0562	Up

**Fig. 4:**
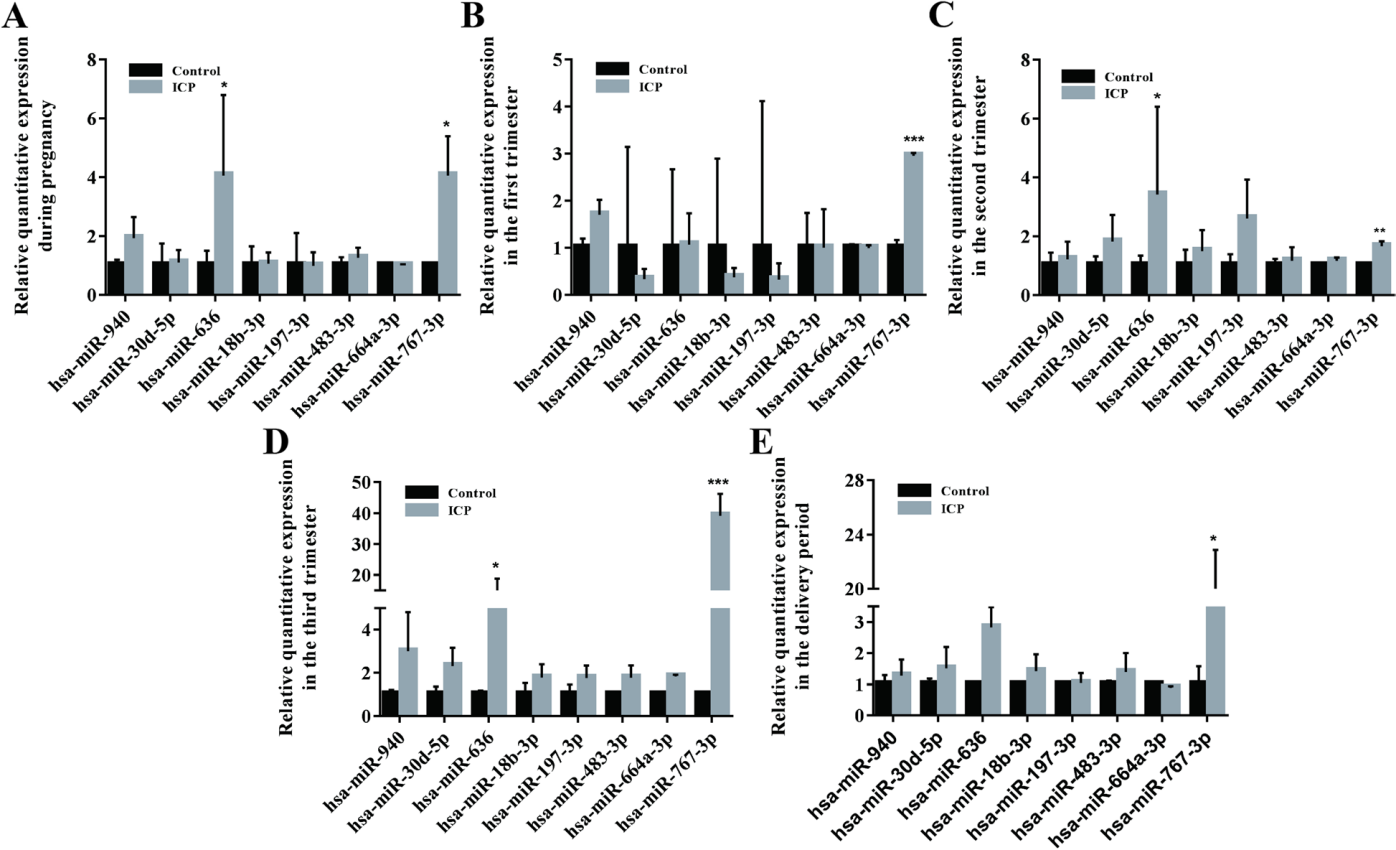
Validation of differentially expressed plasma-derived exosomal miRNAs at the plasma level. **A** Dynamicchangesof differential expressed miRNAs in plasma of ICP patients and healthy pregnant women throughout pregnancy; Changes of differential expressed miRNAs in the plasma of ICP patients and healthy pregnant women in thefirst trimester (**B**), second trimester (**C**), third trimester (**D**) and delivery (**E**). Con, healthy pregnant women; Case, ICP patients. *P < 0.05; *** *P* < 0.001

We used real-time PCR to detect the expression of miRNAs at various stages of T1, T2, T3, and T4 in healthy pregnant women and ICP patients in whole plasma.The results showed that only hsa-miR-636 (*p* < 0.05) and hsa-miR-767-3p (*p* < 0.05) were differentially expressed between the ICP group and the control group, while hsa-miR-940 (*p* = 0.46) showed an increasing trend in the ICP group compared with the control group (Fig. 4A). During the first trimester (T1), only the expression of hsa-miR-767-3p (*p* < 0.001) was significantly increased in the ICP group (Fig. 4B). During the second trimester (T2), only hsa-miR-636 (*p* < 0.05) and hsa-miR-767-3p (*p* < 0.01) were significantly increased in the ICP group (Fig. 4C). Likewise, only hsa-miR-636 (*p* < 0.05) and hsa-miR-767-3p (*p* < 0.001) were significantly increased in the ICP group during the third trimester (T3) (Fig. 4D). And during the delivery period (T4), only the expression of hsa-miR-767-3p (*p* < 0.05) was significantly increased in the ICP group (Fig. 4E).

### Differential expression of hsa-miR-940, hsa-miR-636, and hsa-miR-767-3p at the placental level

**Fig. 5:**
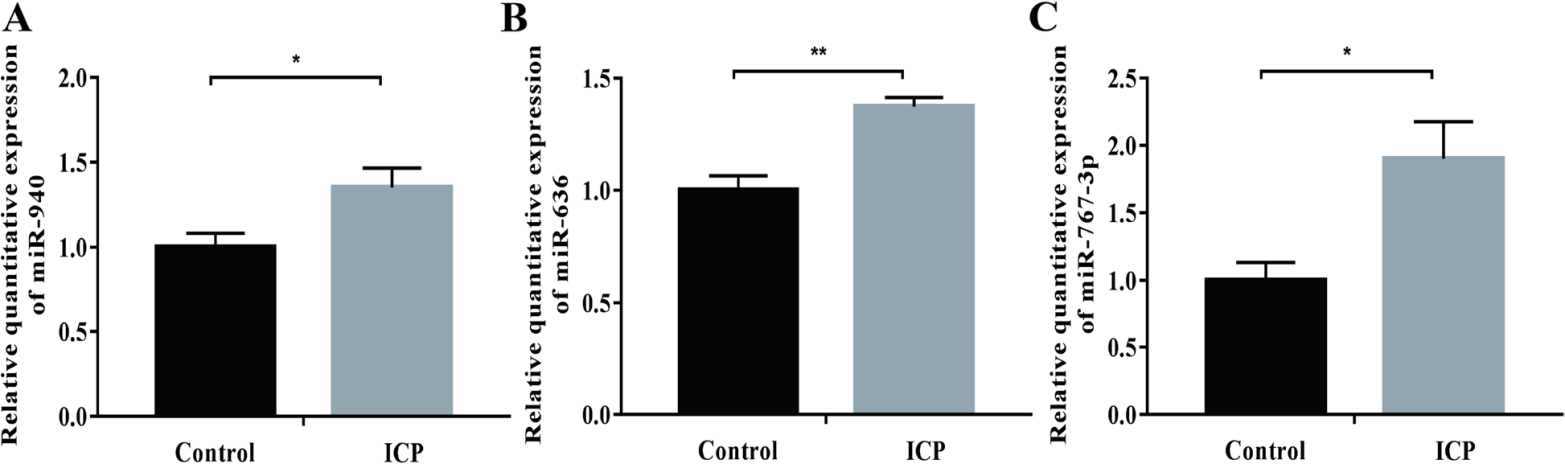
Differential expression of hsa-miR-940, hsa-miR-636 and hsa-miR-767-3p at the placental level. The levels of hsa-miR-940 (**A**), hsa-miR-636 (**B**) and hsa-miR-767-3p (**C**) in ICP and controls. *P < 0.05; **P < 0.01

Subsequently, the expression of these three exosomal miRNAs (hsa-miR-940, hsa-miR-636, and hsa-miR-767-3p) at the placental level was differentially expressedas determined by the real-time quantitative PCR assay. We discoveredthat hsa-miR-940 (*p* < 0.05), hsa-miR-636 (*p* < 0.01) and hsa-miR-767-3p (*p* < 0.05) were significantly upregulated in ICP patients compared with controls (Fig. 5A-C).

### Differential expression of hsa-miR-940, hsa-miR-636, and hsa-miR-767-3p atthe cellular level

**Fig. 6:**
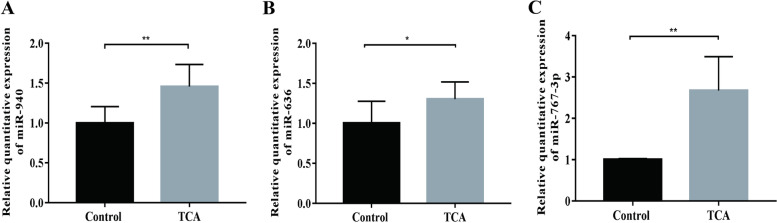
Differential expression of hsa-miR-940, hsa-miR-636 and hsa-miR-767-3p at the cellular level. Expression levels of hsa-miR-940 (**A**), hsa-miR-636 (**B**) and hsa-miR-767-3p (**C**) in TCA and control groups. Control, HTR8/SVneo cells; TCA, cholestatic cell model; *P < 0.05; **P < 0.01

The cells were divided into two groups: HTR8/SVneo cells that had been treated with sodium taurocholate (TCA) and HTR8/SVneo cells withthe blank control group. Real-time quantitative PCR was used to assess the expression of three exosomal miRNAs (hsa-miR-940, hsa-miR-636, and hsa-miR-767-3p) at the cellular level. The results showed that hsa-miR-940 (*p* < 0.01), hsa-miR-636 (*p* < 0.05), and hsa-miR-767-3p (*p* < 0.01) were significantly up-regulated in the TCA-treated group compared with the control group (Fig. 6A-C).

### Predictive value of hsa-miR-940, hsa-miR-636, and hsa-miR-767-3p at the plasma level

**Fig. 7:**
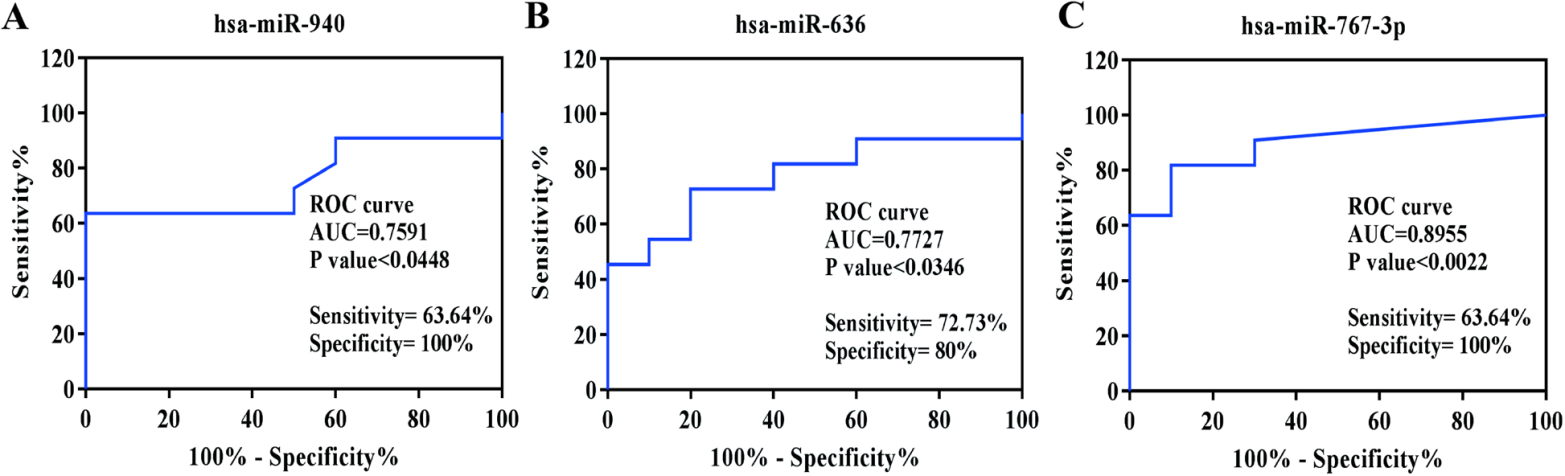
Predictive value of hsa-miR-940, hsa-miR-636, and hsa-miR-767-3p at the plasma level. **A** ROC analysis of hsa-miR-940 (**B**) ROC analysis of hsa-miR-636 (**C**) ROC analysis of hsa-miR-767-3p

To evaluate the likelihood of being diagnosed with ICP, we plotted ROC curves and calculated the AUCs of hsa-miR-940, hsa-miR-636, and hsa-miR-767-3p, respectively. As shown in Fig. 7A-C, the AUCs of hsa-miR-940, hsa-miR-636 and hsa-miR-767-3p were, respectively, 0.7591, 0.7727 and 0.8955, while the sensitivity were 63.64%, 72.73% and 68.64%, and the specificity were 100%, 80% and 100%, respectively. This indicates that hsa-mir-940, hsa-mir-636, and hsa-mir-767-3p have important diagnostic value in predicting the risk of ICP.

## Discussion

Our study has found that the expression levels of hsa-miR-940, hsa-miR-636, and hsa-miR-767-3p in plasma-derived exosomes of ICP were significantly higher than those of the healthy control group. Similarly, the expression levels of these three miRNAs were significantly up-regulatedat the cellular, placental, and plasma levels. This indicates that the analysis and detection of plasma-derived microRNAs in the context of ICP is feasible, and maternal plasma-derived microRNAs have the potential to become non-invasive biomarkers for the diagnosis of ICP.

There are multiple factors involved in the development of ICP, and its pathogenesis is unclear [26–28]. Even though ICP is a benign disease state for the mother, ICP can lead to many adverse pregnancy outcomes, such as preterm birth, intrauterine fetal distress, stillbirth, and so on [29]. We discovered that the gestational week was significantly shorterin ICP patients than in healthy pregnant womenin the current study, indicating that ICP patients are at potential risk of preterm delivery. Early diagnosis and prognosis of ICP patients are therefore of essential clinical significance. Currently, the clinical diagnosis of ICP is largely based on the corresponding clinical symptoms [30] and laboratory criteria [31]. These standards, however, do not serve as the gold standard for diagnosis because of their flaws. Therefore, further studies of the possibility of early sensitive molecules that might occur in the ICP environment are necessary, as well as screening for sensitive and specific biomarkers. In addition, the diagnosis of ICP is generally in the second and third trimesters of pregnancy, but by analyzing the plasma exosomes of ICP patients, we found that miRNAs were specifically expressed in the first trimester, second trimester, third trimester, and delivery, which may provide new research findings for the diagnosis of ICP.

MicroRNAs are involved in many physiological processes, such as cell growth, energy metabolism, apoptosis, infection, and immunity, and are essential for maintaining the homeostasis of the body [32]. Plasma exosome-derived miRNAs are more stable than plasma-derived miRNAs due to the protection of phospholipid bilayer, and miRNAs in exosomes have higher tissue-derived specificity, thus exosomal miRNAs have greater potential in disease diagnosis [33]. Devor et al. found that exosome-derived miRNA patterns in the first trimester of pregnancy differed between women with preeclampsia and those normal pregnancies and could be used for early preeclampsia diagnosis [34]. Nairet al. used a retrospective case–control study to explore exosome-derived miRNAs and found that the expression of hsa-miR-92a-3p, hsa-miR-16–2-3p, and hsa-miR-1910-5p was significantly upregulated in gestational diabetes and their expression changed with increasing maternal BMI [35]. Besides, it has been reported that the expression levels of some exosomal miRNAs in the urine of ICP patients are significantly higher than those of normal controls, indicating that exosomal miRNAs have potential as non-invasive diagnostic urinary biomarkers for ICP [36]. However, there are few studies on the comprehensive analysis of plasmicexosomal miRNA expression profiles in ICP patients.

In this study, the Agilent miRNA array and RT-qPCR were used to compare the relative expression of plasma exosome-derived miRNAs from ICP patients and healthy pregnant women at different stages of pregnancy (early, middle, late pregnancy, and delivery) to identify the unique microRNA signatures in plasmic exosomes of ICP patients, overcoming the unfavorable conditions by obtaining dynamic plasma and placental tissue specimens during pregnancy. We found that hsa-miR-940, hsa-miR-636, and hsa-miR-767-3p were differentially expressed in both plasma exosomes and plasma. Further validation at the placental tissue level and cellular level revealed that hsa-miR-940, hsa-miR-636, and hsa-miR-767-3p were also significantly upregulated in ICP patients.

Hsa-miR-940 is a non-coding RNA located on chromosome 16p13.3, which mainly affects the stability and translation of target protein-coding genes by binding to the 3' untranslated region, thereby participating inpost-transcriptional regulation of gene expression in multicellular organisms [37]. It has been demonstrated that hsa-miR-940 is expressed unbalanced in many diseases such as ovarian cancer [38], breast cancer [39], gastric cancer [40], etc. Zhang et al. found that miR-940 was highly expressed in the villi of early abortions and was capable of inhibiting the proliferation of trophoblasts by targeting ZNF672, leading to early abortions [41]. Cao et al. found that miR-940 regulates the inflammatory response of osteoarthritic chondrocytes by targeting MyD88 [42]. However, the expression of miR-940 in some cancers remains controversial. Liu et al. discovered that downregulation of plasma miR-940 expression in gastric cancer [40], while Yuan et al. found that high expression of miR-940 inhibited hepatocellular liver cancer growth and was correlated with patients' prognoses [43]. In the present study, our bioinformatics analysis showed that plasma exosome-derived miR-940 was significantly upregulated in ICP patients at early, mid, and late pregnancy as well as at delivery, and this trend was also validated at the plasma, placental, and cellular levels.In addition, the AUC of miR-940 was 0.7591, and its sensitivity and specificity were 63.64% and 100%, respectively, suggesting that the upregulation of this exosomal miRNA may become a new biomarker for detecting ICP.

Hsa-miR-636 is a miRNA located on chromosome 17q25.1. It is known to play a role in the occurrence and development of various tumors such as lung cancer [44], prostate cancer [45], etc. Besides, it has been reported that hsa-miR-636 targets cyclin-dependent kinase 6 (CDK6) and B-cell lymphoma factor 2 (Bcl-2), and can inhibit the survival of cervical cancer cells by targeting CDK6/Bcl-2 [46]. In addition, hsa-miR-636 can promote the proliferation of bladder cancer cells by reducing the expression of Kruppel-like factor 9 (KLF9) on the 3’UTR of its mRNA [47]. Although hsa-miR-636 hasshown to play a function in either promoting or inhibiting the formation and progression of various cancer, its expression in ICP and its effect on prognosis have yet to be studied.In our study, we found that plasma exosome-derived hsa-miR-636 was significantly up-regulated in the context of ICP, and its AUC was 0.7727, indicating that hsa-miR-636 has a certain value in the diagnosis of ICP.

Hsa-miR-767 has two types: miR-767-3p and miR-767-5p, among which hsa-miR767-3p is generally considered to be the representative of miRNA-767. Currently, the majority of hsa-miR767-3p-related research is being conducted in the field of cancer research [48]. Wan et al. found that miR-767-3p inhibited lung adenocarcinoma cell proliferation, migration, and invasion by targeting CLDN18 [49]. A study conducted by Zhang et al. showed that miR-767 could function as a tumor promoter in melanoma by targeting CYLD [50]. However, the underlying molecular mechanism of hsa-miR767-3p in the context of ICP has not been thoroughly investigated. Here, we found five genes that hsa-miR767-3p targets. Among them, JARID2 is expressed tissue-specifically and is a regulator of histone methyltransferase complexes that play important roles in embryonic development, including heart and liver development, neural tube fusion process, and hematopoiesis [51]. Furthermore, the AUC of hsa-miR767-3p was 0.8955, which was the largest among the three microRNAs we validated, indicating that hsa-miR767-3p has a high value for diagnosing ICP.

The innovation of this paper is that we are the first one who focused on the comprehensive analysis of the expression profiles of plasmic exosomal miRNAs in ICP patients. Our findings testify that hsa-miR-940, hsa-miR-636, and hsa-miR-767-3p may be potential biomarkers for enhancing the diagnosis of ICP. Besides, we found miRNA specific expression at T1, T2, T3, and T4 different stages through plasma exosome analysis. Given that ICP is now typically diagnosed in the middle and late stages of pregnancy, this finding may provide research findings for the diagnosis of ICP. While the limitation of this study is that this is an exploratory study, the functional roles of these three miRNAs in the emergence of ICP are still unknown. Furthermore, only a small number of samples were evaluated in this study due to difficult specimen collection conditions, and additional research is required to corroborate these findings. Future lines of investigation will build on molecular mechanism studies to look into other diagnostic and/or therapeuticinterventions in ICP disease.

## Conclusions

In conclusion, we identified three differentially expressed miRNAs in theplasmic exosomes of ICP patients and assumed that hsa-miR-940, hsa-miR-636, and hsa-miR-767-3p may be potential biomarkers for enhancing the diagnosis and prognosis of ICP.

## Supplementary Information

**Additional file 1: **Fig 1C. In order to improve the clarity and conciseness of the presentation, the main paper shows the blot after cutting. The original, uncropped blot have been uploaded to the additional file.

**Additional file 2: Table S1. **Sequences of the reverse transcription quantitative polymerase chain reaction primers used.

## Data Availability

All data generated or analyzed during this study are included in this published article. The Agilent miRNA array data were deposited in the Gene Expression Omnibus (https://www.ncbi.nlm.nih.gov/geo/query/acc.cgi?acc=GSE210764; GEO accession number GSE210764).

## Abbreviations

ICP: Intrahepatic cholestasis of pregnancy
TBA: Total bile acids
BMI: Body mass index
ALT: Alanine aminotransferase
AST: Aspartate aminotransferase
