# Supplementary material for: Unique microRNA expression profiles in plasmic exosomes from intrahepatic cholestasis of pregnancy
Source: BMC Pregnancy Childbirth. 2023 Mar 7;23:147. doi: 10.1186/s12884-023-05456-1 (PMC9990296; doi:10.1186/s12884-023-05456-1)
Supplement: Supplementary file 1 — Additional file 1: Fig 1C. In order to improve the clarity and conciseness of the presentation, the main paper shows the blot after cutting. The original, uncropped blot have been uploaded to the additional file. [file 12884_2023_5456_MOESM1_ESM.docx]

Fig1C. In order to improve the clarity and conciseness of the presentation, the main paper shows the blot after cutting. The original, uncropped blot have been uploaded to the additional file.
