# Supplementary material for: Unique microRNA expression profiles in plasmic exosomes from intrahepatic cholestasis of pregnancy
Source: BMC Pregnancy Childbirth. 2023 Mar 7;23:147. doi: 10.1186/s12884-023-05456-1 (PMC9990296; doi:10.1186/s12884-023-05456-1)
Supplement: Supplementary file 2 — Additional file 2: Table S1. Sequences of the reverse transcription quantitative polymerase chain reaction primers used. [file 12884_2023_5456_MOESM2_ESM.doc]

| **Table S1**  Sequences of the reverse transcription quantitative polymerase chain reaction primers used. | |
| --- | --- |
| **Name** | **Sequence** |
| Has-miR-940 | Forward: 5'-TGACAGAAGAGAGTGAGCAC-3' Reverse: 5'- GCAGGGTCCGAGGTATTC -3' |
| Has-miR-636 | Forward: 5'-GCTCAAGAAAGCTGTGGGAAA-3' Reverse: 5'- GCAGGGTCCGAGGTATTC -3' |
| Has-miR-767-3P  U6 | Forward: 5'- TTCGCAGGAGAGATAGCGCCA -3'  Reverse: 5'-GCAGGGTCCGAGGTATTC-3'  Forward: 5'-TGGAACGCTTCACGAATTTGCG-3'  Reverse: 5'-GGAACGATACAGAGAAGATTAGC-3' |
